# Birth Prevalence of Sickle Cell Disease in India: A Systematic Review and Meta-Analysis

**DOI:** 10.3390/ijns12010010

**Published:** 2026-02-25

**Authors:** Emine A. Rahiman, Rajendra Prasad Anne, Rajasekharan P. Warrier

**Affiliations:** 1 Department of Pediatric Oncology, Kasturba Medical College, Manipal Academy of Higher Education (MAHE), Manipal 576104, Karnataka, India; emine.rahiman@manipal.edu; 2 Department of Neonatology, Kasturba Medical College, Manipal Academy of Higher Education, Manipal 576104, Karnataka, India; 3 Pediatric Hematology Oncology, Ochsner Children’s Hospital, New Orleans, LA 70121, USA; rwarrier@ochsner.org

**Keywords:** under-5 mortality, tribal health, SDG 4, preventive care, hydroxyurea

## Abstract

Newborn screening helps identify sickle cell disorder (SCD) early and to promptly initiate effective measures. It is estimated that India accounts for approximately 16% of global annual births with SCD. Multiple reports of screening for SCD in India have emerged in the last decade. Our aim was to pool the birth prevalence of SCD and sickle cell trait (SCT). A systematic review of published evidence on nontargeted, universal screening for SCD or SCT in newborns was performed (16 studies). The pooled prevalence of SCD was 1100 per 100,000 (10 studies, 88,276 neonates, 95% CI: 432, 1768), while that of SCT was 9639 per 100,000 (7 studies, 72,702 neonates, 95% CI: 6283, 12,995) in endemic regions. Limited data exist from nonendemic regions. Only three studies had data on follow-up and confirmatory genetic diagnosis. Sparse data exist on cost-effectiveness, long-term follow-up, and the impact of early screening on mortality. Concerted ongoing efforts in the identification of the burden are needed. The needs of the hour are universalization of NBS, integration into existing health systems, and maintenance of birth cohorts with early introduction of penicillin prophylaxis, hydroxyurea, parental education, appropriate immunization, and continued follow-up by an experienced medical team.

## 1. Introduction

Sickle cell disease is the most common monogenic disorder in India [1], with inherent acute and chronic complications if undiagnosed and untreated. The Global Burden of Diseases (GBD) report on sickle cell disorders (SCDs) estimated approximately 82,500 births with sickle cell disorders (homozygous sickle cell, compound heterozygous HbS-thal, hemoglobin SC disease, compound heterozygous HbS-beta+) in India, with approximately 15,600 under-5 deaths attributable to these disorders, making them the 10th most common cause of under-5 mortality in the country [2]. They also found that India, along with the western and central parts of Africa, has the highest concentration of sickle cell disease disability burden. The prevalence of sickle cell trait (SCT) is as high as 35% in the endemic tribal population to nonexistent in other parts of the country [3]. The endemic regions in the country are South Gujarat, Maharashtra, Madhya Pradesh, Chhattisgarh, and western Odisha, with a smaller focus in Andhra Pradesh, Karnataka, northern Tamil Nadu, and Kerala [3].

The diagnosis and management of SCD have not been ideal in India due to regional, economic, and educational disparities. Targeted and protocolized follow-up exists only in pockets of high prevalence. National Family Health Survey **5** (NFHS-5) data show that the tribal population, which accounts for 8.6% of India’s population, has high under-5 mortality (52 per 1000 live births compared to 42 overall) and infant mortality (43 per 1000 live births versus 35.2 overall) [4]. The key reasons are a lack of systems for the early diagnosis of health issues, remote locations reducing access to good-quality healthcare services, an inequitable distribution of resources, and resistance to modern medical care.

Presently, under-5 mortality in India is 42 per 1000, and with current trends of improvement, it is unlikely that India will reach the SDG 2030 target (25 per 1000). Universal NBS is one of the tools to improve under-5 mortality. Early diagnosis of treatable disorders helps prevent progression and damage due to the disease. NBS helps in implementing preventive measures, providing opportunities for parental education, ensuring compliant follow-up, and helping reduce infant and child mortality.

The Indian health system is heterogeneous, with a complex mixture of government and private agencies [5]. It is a three-tier system (primary, secondary, tertiary) with individual states being primarily responsible. Often, due to constraints in the public setup, private centers must be relied upon. The newborn screening program in India is in its early stages, with only three states (Goa, Kerala, and Chandigarh) having state-run programs [6,7]. Although there is public medical consensus on the need to screen, no national strategy or direction exists to implement a national program. The challenges faced are lack of universal acceptance, low awareness, communication on reports and initiation of treatment, lack of robust follow-up, and quality control of laboratories conducting the tests [6]. In a highly populated country, the implementation of newborn screening and the creation of disease-specific birth cohorts, along with the provision of comprehensive preventive and therapeutic healthcare services for affected individuals, will help reduce disease-related morbidity and mortality.

It is heartening to see the significant leaps made in the last decade in India, particularly with targeted screening of babies born to mothers with sickle cell disorders [8,9] and those from endemic regions [10]. Universal newborn screening (NBS) is being implemented in parts of South Gujarat, Maharashtra, Madhya Pradesh, Chhattisgarh, Odisha, and Tripura. A screening of 18,003 babies conducted in 2018 identified 2944 as sickle cell carriers and 300 as having SCD [11]. The Indian Council of Medical Research (ICMR) is conducting a screening of 60 million people in 17 identified tribal-dominated states. Screening of 63,536 newborns conducted from 2019 to 2024 in endemic regions diagnosed 7275 babies (11.4 percent) as sickle cell carriers and 569 babies (0.9 percent) as SCD [12,13]. The increasing availability of high-performance liquid chromatography (HPLC) machines in the government sector makes them accessible for use in screening programs.

It is imperative to quantify the burden of SCD in newborns in our country (i.e., the birth prevalence). The quantity of effort required to eliminate the disease by 2047 will depend on the accurate assessment of the numbers of patients who need care. We collated all the published evidence on newborn screening conducted to identify SCD and SCT in India and pooled the birth prevalence data for these conditions.

## 2. Materials and Methods

### 2.1. Search Strategy and Selection Criteria

The protocol for this systematic review and meta-analysis was prospectively registered in the Open Science Framework database [14] and can be accessed at https://osf.io (accessed on 13 January 2026). We followed the PRISMA (Preferred Reporting Items for Systematic Reviews and Meta-Analyses) reporting guidelines [15].

We searched the PubMed, Embase, Scopus, and Web of Science databases from their inception until 7 November 2025. We designed our search strategy to identify all studies on newborn screening from India. We also searched for citations and reviewed the references of the eligible articles to identify additional studies. Gray literature and unpublished data were identified by searching Google, Google Scholar, government websites, dissertation websites (Shodhganga, open-access theses and dissertations) and preprints (medRxiv.org). We contacted researchers who were working on these topics. The search strategy is shown in Table A1.

### 2.2. Inclusion and Exclusion Criteria

All observational studies from India that assessed one or more of the three outcomes of interest, i.e., the prevalence of SCD or SCT, screen-positivity rates, or burden or cost, were included. A study was considered eligible for inclusion if it was a cross-sectional prevalence study (i.e., all neonates born or admitted to the unit or from a particular locality were screened for SCD). We excluded studies where the authors did not state the sample size and the number of subjects who tested positive, where targeted newborn screening was performed (i.e., babies born to mothers with SCD or SCT were screened), and studies where screening was performed beyond the neonatal period. Conference abstracts without formal publication, systematic reviews, meta-analyses, letters or correspondence without original data, guidelines, and multinational studies where separate data for Indian neonates could not be obtained were also excluded.

### 2.3. Outcomes

The primary outcome of interest was the prevalence of SCD. The diagnostic criteria used were the presence of very high levels of HbS (>90%) for homozygous sickle cell disease and HbS of 30–40% for the sickle cell trait. The secondary outcomes included burden in different settings (endemic and nonendemic, tribal, and nontribal), as well as the associated costs.

### 2.4. Data Handling

We exported the articles retrieved from the databases to Rayyan software [16]. After removing the duplicates, two reviewers (RPA and EAR) assessed the titles and abstracts for potentially eligible articles in a blinded manner, and any discrepancies were resolved by mutual discussion and involvement of the third reviewer (RPW). We retrieved full texts of eligible articles and extracted the following data: study identification details; study and population characteristics including study setting (tribal or nontribal), place of study (endemic or nonendemic), duration, study design (prospective or retrospective), inclusion and exclusion criteria, details of screening tests including the sampling method, analytical methods used (high-performance liquid chromatography (HPLC) or electrophoresis), and diagnostic criteria, details of confirmatory testing–sampling strategy for screen-positive neonates, and criteria for confirming the diagnosis of SCD, and data on follow-up. Duplication of the dataset was assumed when the study duration and place of study were similar or overlapping. Studies that were performed over the longest period were included.

### 2.5. Quality Assessment

Two reviewers independently assessed the quality of the included studies using the Joanna Briggs Institute tool for assessing the quality of prevalence studies (https://jbi.global/critical-appraisal-tools accessed on 13 January 2026). The quality was rated lower when the neonates were excluded inappropriately, the sampling strategy did not include consecutively born neonates, the sample size was inadequate (<4226, assuming 1 + 0.3% prevalence of SCD) [17], population characteristics were not described in detail, analytical methods used were inappropriate, or when the diagnostic criteria were not as per guidelines. The overall risk of bias for a study was rated high if at least one domain was at high risk of bias. We included all eligible studies in the meta-analysis, irrespective of quality.

### 2.6. Statistical Analysis

Since the prevalence of SCA is below 0.005 (approximately 0.001 to 0.002 in previous studies), the Freeman–Tukey double-arcsine transformation method was employed to stabilize the variance [18]. SCT has a prevalence above 0.005, and we used a random-effect model (DerSimonian and Laird method) to account for within-study and between-study variances [19]. The overall pooled prevalence was not estimated, as the prevalence rates for SCD and SCT varied widely in endemic versus nonendemic regions and tribal versus nontribal populations. The prevalence is reported as the number of cases per 100,000 population screened, in accordance with the GBD standards [2]. We used R statistical software (meta package) version 4.5.1 for Windows (Copyright (C) 2025 The R Foundation for Statistical Computing) for meta-analysis. Heterogeneity was assessed using Cochran’s Q, I2, τ2, and prediction intervals, as well as the variability of estimates across the studies in the forest plots and the variance of the pooled estimates [20]. We planned to assess publication bias using funnel plots, Doi plots, and the LFK index [21]. We planned a sensitivity analysis after excluding studies at high risk of bias.

## 3. Results

### 3.1. Search Results

**Figure 1:**
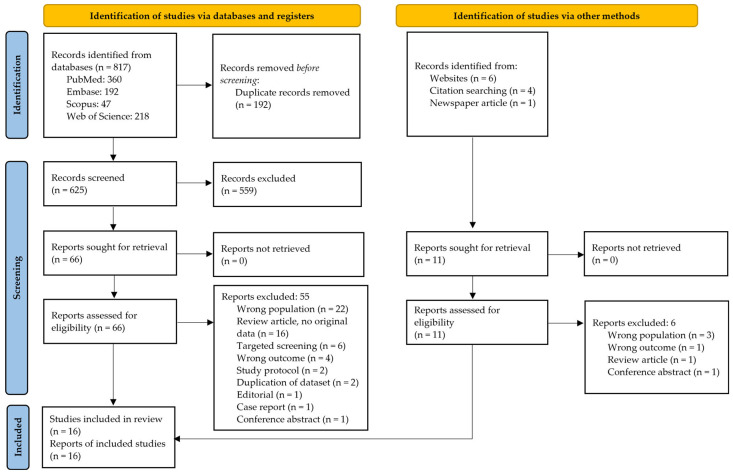
PRISMA flow diagram.

The database search yielded 625 unique titles. Ten additional titles were found through gray literature and citation searches. In sum, 77 full-text articles (66 from databases and 11 from other sources) were evaluated for eligibility, and 16 studies (11 from databases and 5 from other sources) were included in the meta-analysis [12,22,23,24,25,26,27,28,29,30,31,32,33,34,35,36]. The PRISMA flow diagram is shown in Figure 1. The excluded studies are shown in Table A2.

### 3.2. Characteristics of Included Studies

Ten studies were conducted in endemic regions [12,23,24,25,26,28,32,34,35,36], and six were conducted in nonendemic regions [22,27,29,30,31,33]. The characteristics of the included studies are provided in Table 1. Most studies were conducted in the state of Maharashtra [24,26,32,33,36]. Other studies were from the states of Telangana [27,29], Tripura [30], Rajasthan [31], Odisha [23], Chhattisgarh [28], West Bengal [22], and Gujarat [24]. Three studies were conducted across different states [12,34,35]. Dried blood spot testing was the preferred method of sample collection [12,22,24,25,27,28,29,31,33,34,35,36], and HPLC was used for analysis in all studies. The HemoTypeSC assay was used in two studies. Confirmatory genetic testing was performed in only three studies [12,30,31]. Four studies reported the types of SCD. Sickle beta-thalassemia was reported in three studies (27 newborns), and HbS-HbD Punjab was reported in 1 newborn in one study. Follow-up data were mentioned in three studies [25,28,35]. Details on genetic diagnosis were available in four studies [25,30,31,35]. Treatment details were reported in four studies [25,28,31,35].

### 3.3. Quality Assessment

**Figure 2:**
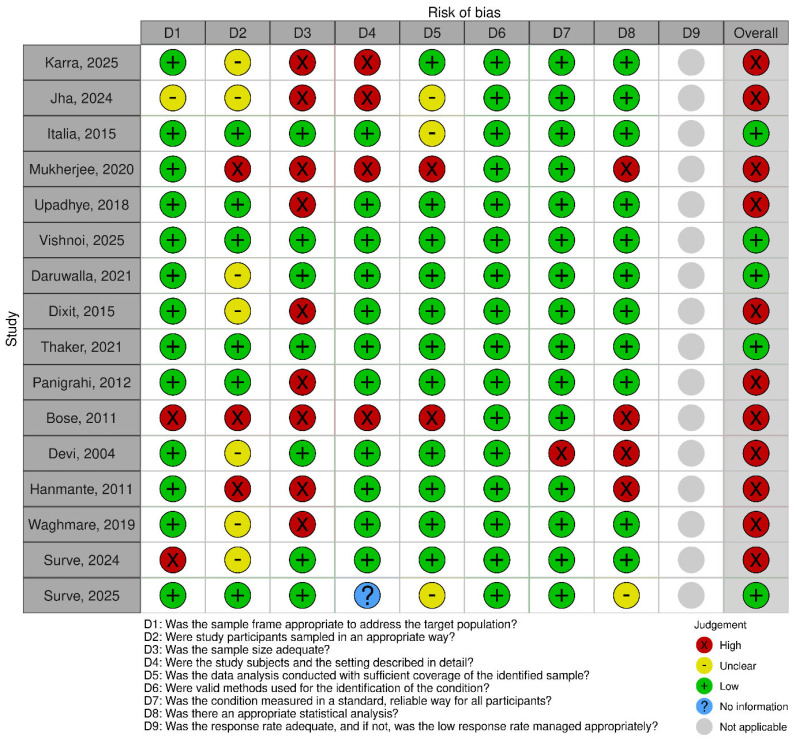
Quality assessment [13,22,23,24,25,26,27,28,29,30,31,32,33,34,35,36].

On quality assessment (Figure 2), five studies were identified to be at low risk of bias [9,12,25,33,35]. The primary reasons for increased risk of bias in other studies were inadequate sample size (*n* = 8 studies), inadequate description of study participants (*n* = 4), and inappropriate statistical analysis (*n* = 4).

### 3.4. Meta-Analysis

**Figure 3:**
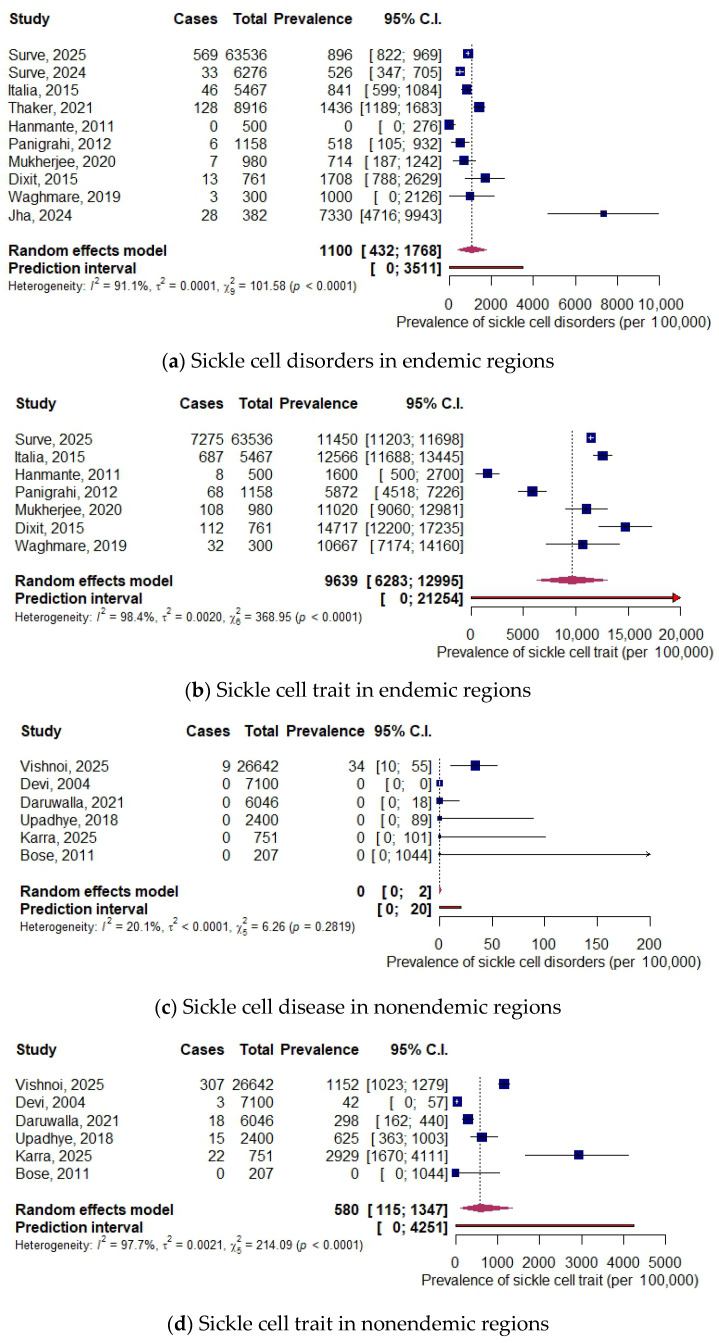
Forest plots for prevalence in endemic and nonendemic regions [13,22,23,24,25,26,27,28,29,30,31,32,33,34,35,36].

In endemic regions, the prevalence of SCD was 1100 per 100,000 neonates screened (10 studies, 88,276 neonates, 95% CI: 432, 1768), while that of SCT was 9639 per 100,000 neonates screened (7 studies, 72,702 neonates, 95% CI: 6283, 12,95). In nonendemic regions, the prevalence of SCD was close to 0 per 100,000 neonates screened (6 studies, 43,146 neonates, 95% CI: 0, 2), while that of SCT was 580 per 100,000 neonates screened (6 studies, 43,146 neonates, 95% CI: 115, 1347). The forest plots are shown in Figure 3.

**Figure 4:**
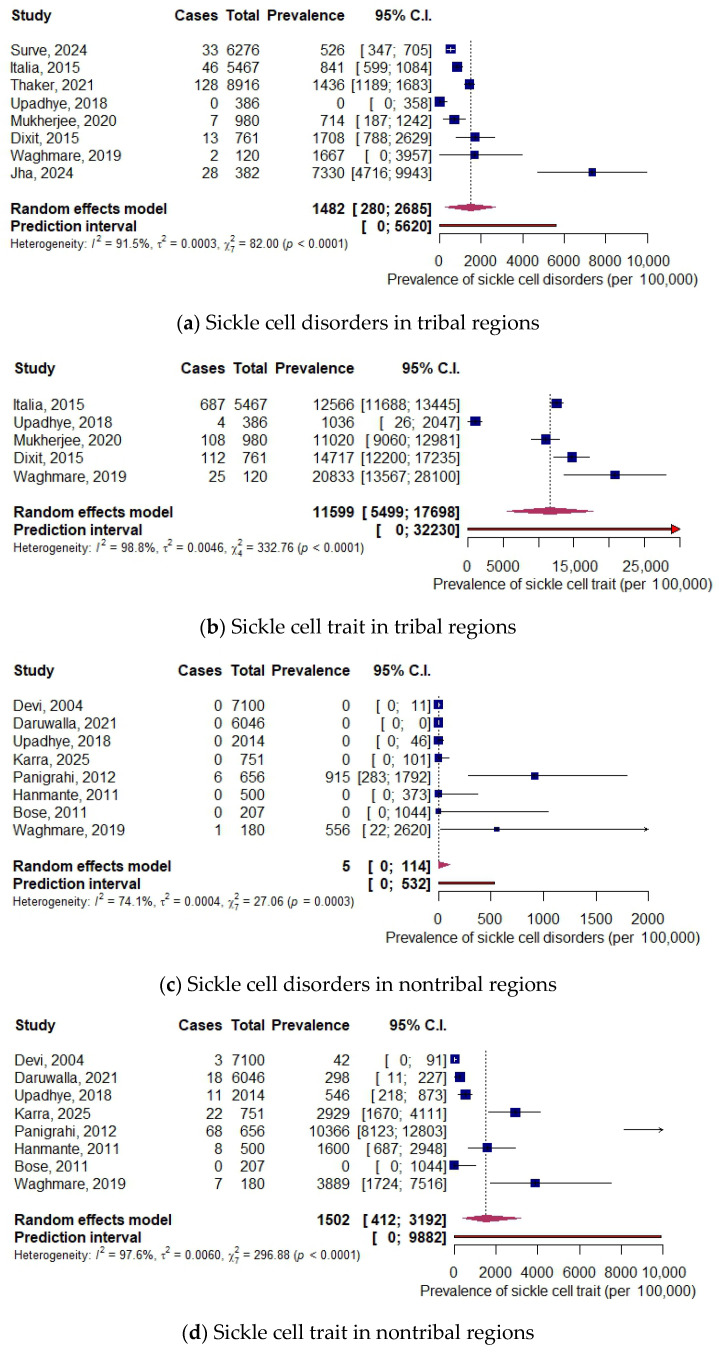
Forest plots for prevalence in tribal and nontribal populations [22,23,25,26,27,29,30,32,33,34,35,36].

The tribal population had an SCD prevalence of 1482 per 100,000 neonates (8 studies, 23,288 neonates, 95% CI: 280, 2685) and an SCT prevalence of 11,599 per 100,000 neonates (5 studies, 7714 neonates, 95% CI: 5499, 17,698). The birth prevalence in nontribal regions was 5 per 100,000 neonates for SCD (8 studies, 17,454 neonates, 95% CI: 0, 114) and 1502 per 100,000 neonates for SCT (8 studies, 17,454 neonates, 95% CI: 412, 3192). The forest plots are shown in Figure 4.

The heterogeneity was low for SCD prevalence in nonendemic regions and high for other outcomes. We did not perform subgroup analysis, meta-regression, sensitivity analysis, or publication bias assessment due to the inadequate number of studies. We could not analyze the outcome of cost, as no study reported data on cost.

We extrapolated these data to estimated annual births in India in 2024 (based on a population estimate of 1.44 billion and a crude birth rate of 16 per 1000 population) [37]. Assuming that the tribal population constitutes 8.6% of the population [4], with a total fertility rate of 2.5 [38] for the tribal population and 2 for the nontribal population [4], there were likely 24.2 per 100,000 births in tribals and 206.2 per 100,000 births in the nontribal population. The number of newly born babies with sickle cell disorders is estimated to be approximately 35,800 in the tribal population and 1030 in nontribal populations (Table 2).

## 4. Discussion

Our systematic review and meta-analysis summarizes the data on the birth prevalence of sickle cell disease in India. While endemic regions had a 1100 per 100,000 birth prevalence of sickle cell disorders and 9640 per 100,000 of sickle cell trait, the prevalence in nonendemic regions was negligible, with SCD prevalence close to ‘0’ and SCT prevalence of 580 per 100,000.

The effectiveness of NBS is proven in the history of medicine. Early diagnosis by NBS, vaccination against pneumococcal and *Haemophilus influenzae*, penicillin prophylaxis [39], and early introduction of hydroxyurea help prevent deaths and improve quality of life in children with SCD. Other benefits include prompt clinical intervention for infection, early education and awareness of parents about signs and symptoms of illness in early childhood, and prenatal diagnosis in subsequent pregnancies [40,41,42]. The universal NBS program also helps diagnose multiple life-threatening ailments in one go. NBS for sickle cell disorders was initiated in the USA in the 1960s and started with targeted population screening, evolving into identifying public health laboratories, conducting multicenter studies, trials on prophylactic penicillin use, and final recommendation of universal NBS in 1987. Since 2006, NBS has been universally implemented for common forms of SCD (HbSS, Hb SC, and Hb S/β thalassemia) in all states of the USA. The screening, reporting, and referral of other hemoglobinopathies, however, is widely variable across states [43].

The strong effect of NBS on reducing mortality in hemoglobinopathies was demonstrated in the US, where from 1975 to 1985, 84,663 newborns were screened, irrespective of ethnicity and background. They identified SCD in 1 in 951 births and significant thalassemia syndrome in 1 in 4233 newborns. Significantly, the overall mortality rate when sickle cell disease was diagnosed in the newborn period was only 1.8%, while it was 8% when diagnosed beyond 3 months of age [41]. In the USA, with the implementation of universal NBS for SCD and the initiation of penicillin prophylaxis, mortality has been reduced by 50% in affected children aged 1 to 4 years [41]. It was also noticed that overall life expectancy has increased from a median of 14.3 years to between 42 and 53 years in males and 46 and 58.5 years in females [41].

Efforts have been scaled up to establish NBSs for SCD in Africa since 2010. Sub-Saharan Africa accounts for 75% of the global prevalence of SCD. There is heterogeneity in practices: in some countries, sample collection occurs at the primary level and is transported to tertiary facilities, while in a few other countries, it is integrated into other national health programs, and in most states, services are available only in tertiary healthcare. It was observed that factors such as government ownership in the form of policy formulation and funding, involvement of healthcare stakeholders in policy formulation, acceptability of NBS among stakeholders, and integration of NCD into existing services were enablers for implementing NBS. They could also establish follow-up, facilitated by preexisting SCD clinics, obtain funding from industry partnerships, and create communication channels for tracing patients [44,45,46,47]. The barriers observed were lack of public awareness, low level of education of mothers, limited supply of diagnostic laboratory materials, epileptic electricity, lack of trained healthcare workers, low access to screening facilities, parental denial of positive results in apparently healthy infants, low accessibility to comprehensive care services, unsuccessful tracking due to wrong contact numbers, and delay in communication channels [45,46,47,48,49]. With global partnerships such as the establishment of a consortium on newborn screening in Africa (CONSA) program with the American Society of Hematology in 2017, increasing success has been achieved in Africa [50]. For India, lessons learnt from other countries where NBS programs are successful are paramount to establish its indigenous NBS program.

In India, concerted efforts to identify SCD-affected children in endemic regions have occurred in the last decade. Over the years, studies have evolved from single-center tertiary hospital-based studies [24,28] to multicentric network creation for screening [13,33,34]. The feasibility and utility of DBS for NBS of SCD is established well beyond doubt. Hence, NBS for SCD could be incorporated in states with ongoing NBS programs. The potential strengths that are identified from included studies in this analysis are (1) availability and use of HPLC, indirectly showing the availability of machines, consumables, and manpower for interpretation, (2) availability of clinics providing comprehensive care in a few endemic regions that can be created as a model to be applied in other regions [13,25], (3) availability of genetic studies for the diagnosis of hemoglobinopathy [9,31,35], (4) fair proportion of follow-up of 70% for confirmation of diagnosis [25,28,35], (5) education of family members [25,35], (6) use of genetic diagnosis for genetic counseling and antenatal diagnosis [35], and (7) use of mobile phones for follow-up and education [35]. The barriers that are globally felt are applicable to India as well, namely lack of public awareness, inconsistent funding, government policy not translating to grassroots changes, tertiary care-centered programs, and nonavailability in primary or secondary level healthcare, and poor channels of follow-up.

Early initiation of hydroxyurea (≤9 months of age) is proven to be safe and effective in the reduction of initial and recurrent episodes of pain, dactylitis, acute chest syndrome, and hospitalization [51]. Less dactylitis, as well as fewer hospitalizations and transfusions, were observed in asymptomatic infants treated with hydroxyurea. Long-term adherence to hydroxyurea effectively reduces the number of emergency department (ED) visits per year, hospital days per year, and the annual average hemoglobin concentration [52]. In studies included in this meta-analysis, one study reported the use of hydroxyurea [35]. A systematic review on the use of hydroxyurea in 3817 Indian patients with SCD (27 studies) showed that HU significantly increased HbF levels (10.9–77.3%), reduced veno-occlusive crisis frequency by 79–93%, and lowered transfusion needs by 50–85%. However, only one study analyzed in that review included children under 2 years of age [53]. Although the recommendation is to start hydroxyurea at 9 months of age, a conservative approach (initiation beyond 2 years of age) and inconsistency in initiating hydroxyurea were observed in India.

Introduction of point-of-care testing (POCT) is one of the key takeaways from the African experience. The most common and available POCTs, including HemoTypeSC, use a qualitative lateral flow immunoassay method to detect the presence of HbA, HbS, and HbC. This is a monoclonal antibody-based test with high accuracy in detecting HbA, HbS, and HbC antigens. Other available instruments are Sickle SCAN and HemeChip. The advantages of POCT are simplicity in performance and interpretation, low cost of device, no requirement of maintenance, electricity, or refrigeration, and the interpretation of test results being visual and qualitative. It is an attractive alternative that can be mass-implemented in low-resource countries at low project cost. The application of POCT in NBS has already been established [54,55,56]. Two studies included in this meta-analysis utilized HemoTypeSC and reported sensitivity and specificity of 98.1% and 99.1% for all possible phenotypes [13,34]. A pilot study looking at the operational feasibility of POCT with training and utilizing primary-level healthcare workers has shown promise in India [54].

The National Sickle Cell Elimination Program (NSCAEM) launched in 2023 aims to cover 70 million people in endemic districts and eliminate SCD by 2047 through improved awareness, early diagnosis, and initiation of prompt preventive care [57]. It is currently one of the flagship programs under the National Ministry of Health and Family Welfare. Although it is not targeted for screening newborn ages, early diagnosis is one of the positive takeaways. India, with its widely heterogeneous demographic characteristics, should initiate the NBS program for SCD tier wise. The strategies from other countries that can be adopted are strong policy advocacy from patient groups, ensuring government funding for the establishment and sustenance of programs, creating comprehensive care centers in nodal centers, using international collaborations, and implementing POCT in peripheral remote regions. The option is to begin with endemic districts with tribal populations, followed by less endemic districts and less affected states, leading to countrywide implementation of the model. Sparse data are currently available for cost-effectiveness newborn screening, genetic counseling, antenatal diagnosis, and POCT. The success of these programs in the USA and UK with significant reductions in mortality and morbidity is a great motivation for our country to support the comprehensive sickle cell program being proposed now [58,59,60].

Our analysis and previous studies indicate that the burden is high in endemic regions, while it is less studied in nonendemic regions. The strengths of this review are a comprehensive picture of birth prevalence across India and the inclusion of tribal/nontribal and endemic/nonendemic populations. However, the study is limited by heterogeneity in the sampling strategy, inadequate sample size, heterogeneity in inclusion criteria, and nonuniform operational definitions in the included studies. Data were not available from many states of the country.

## 5. Conclusions

The data from this analysis show the burden of SCD in India, mostly from endemic/tribal regions. There is an urgent need to quantify the problem in nonendemic/nontribal regions of our country. Establishing birth cohorts and providing comprehensive healthcare will help us understand the effect of early diagnosis on mortality. Universal NBS holds the key for early prompt holistic care.

## Figures and Tables

**Table 1:** Characteristics of included studies.

Author, Year, State	Place of Study, Design and Duration	Inclusion Criteria	Exclusion Criteria	Analytical Methods
Karra, 2025 Telangana [27]	Government Maternity Hospital, Hanamkonda	Term healthy newborns (unclear if consecutive)	Poor sample quality	HPLC
	Prospective study			
	Study duration not specified			
Jha, 2024 Maharashtra [26]	Central India (place of study not specified)	Newborns from various communities (unclear if consecutive)	Not specified	HPLC
	Prospective study			
	January to December 2023			
Italia, 2015 Gujarat [25]	Newborn screening among tribal populations in 4 districts of Gujarat (Valsad, Navsari, Dang, Surat)-10 hospitals	Babies were screened at birth or within 1 month (unclear if consecutive)	Not specified	HPLC using Bio-Rad Laboratories
	Prospective study			
	2 years (exact time not specified)			
Mukherjee, 2020 Maharashtra & Gujarat [34]	Four centers from Maharashtra and Gujarat (Valsad, Jhagadia, Nagpur and Chandrapur)	Newborns from 4 centers of Maharashtra and Gujarat (likely random)	Not specified	HemoTypeSC assay and HPLC (Bio-Rad)
	Prospective study			
	Study duration not specified			
Upadhye, 2018 Tripura [30]	Government Medical College and Govinda Ballav Pant Hospital, Agartala	Universal newborn screening program (consecutive)	Not specified	HPLC (VARIANT TM Bio-Rad) for screening; DNA analysis with covalent reverse dot-blot hybridization (CRDB) for confirmation
	Prospective study			
	3 years (exact time not specified)			
Vishnoi, 2025 Rajasthan [31]	RNT Medical College, Udaipur;	All neonates delivered at the institute (consecutive)	Not specified	HPLC (Bio-Rad VARIANT) for screening; PCR for confirmation
	Prospective study			
	January 2022 to December 2024			
Daruwalla, 2021 Maharashtra [33]	City- Mumbai, setting unclear (details of Hospitals and Clinics not provided)	Newborns whose DBS samples were referred from hospitals and private clinics (likely not consecutive)	Not specified	HPLC (Bio-Rad VARIANT) for screening; Variant II open HPLC system for comparison
	Prospective study			
	January 2016 to November 2018			
Dixit, 2015 Odisha [23]	Kalahandi District Hospital, Bhawani Patna, Odisha	Newborns delivered at Kalahandi District Hospital (likely consecutive)	Not specified	HPLC (Bio-Rad VARIANT)
	Prospective study			
	March 2013 to June 2013			
Thaker, 2021 Gujarat & Madhya Pradesh [35]	Universal newborn screening at Valsad, Navsari, Dang, Surat and Jabalpur Targeted screening at Bharuch	Universal newborn screening at Valsad, Navsari, Dang, Surat and Jabalpur, Targeted screening at Bharuch	Not specified	HPLC (Bio-Rad VARIANT for DBS and Bio-Rad VARIANT 2 for cord blood)
	Prospective study			
	Six years (2010–2016)			
Panigrahi, 2012 Chhattisgarh [28]	Dr BRAM Hospital (JN Medical College, Raipur)	All newborns of women delivered at Dr BRAM Hospital (universal)	Sick neonates admitted to NICU and neonates given blood transfusion	HPLC-sickle short
	Prospective study			
	February 2008 to January 2009			
Bose, 2011 West Bengal [22]	IPGMER, Kolkata	Inborn babies within 3–7 days of life	Not specified	Isoelectric focusing (Perkin Elmer)
	Prospective study			
	2 months (exact dates not specified)			
Devi, 2004 Telangana [29]	Pilot program by CDFD, Hyderabad	Newborn babies between 6 + 2 days	Not specified	Not specified
	Prospective study			
	Exact time not specified			
Hanmante, 2011 Maharashtra [24]	Government Medical College, Aurangabad	Routine deliveries under observation, newborn admitted for other significant illnesses like anemia, fever, etc. CBC performed on newborns with Hb < 14 mg/dL tested with HPLC (not consecutive)	Not specified	HPLC sickle short
	Prospective study			
	January 2009 to November 2010			
Waghmare, 2019 Maharashtra [32]	Regional Hemoglobinopathy Detection and Management Centre, Vidarbha Region	Mothers delivered at Government Medical College; Nagpur were randomly selected	Not specified	HPLC (further details not provided)
	Prospective study			
	2 years (exact time not specified			
Surve, 2024 Maharashtra [36]	Model Rural Health Research Unit (MRHRU), Dahanu in Palghar district, Maharashtra	All newborns (≥28 weeks) delivered at Subdistrict Hospital (SDH), Dahanu and Kasa	Not specified	HPLC-Variant Hb testing system (Bio-Rad)
	Prospective study			
	Duration not specified			
Surve, 2025 Rajasthan, Gujarat, Tamil Nadu, Odisha, Maharashtra and Madhya Pradesh [12,13]	National Institute For Implementation Research on Non-Communicable Diseases in Jodhpur, Society for Education, Welfare and Action-Rural (SEWA-Rural) in Gujarat, the Nilgiris Adivasi Welfare Association (NAWA), Tamil Nadu, ICMR- National Institute for Research in Reproductive Health in Mumbai, ICMR-National Institute of Research in Tribal Health (NIRTH) in Jabalpur, ICMR-Regional Medical Research Centre, Bhubaneswar and ICMR- Centre for Research Management and Control of Hemoglobinopathies (CRHCM) in Chandrapur	All the live-born babies delivered within the facilities of selected regions	Stillbirths and intrauterine fetal deaths	Hb-Variant-II testing system (Bio-Rad Laboratories, Hercules, CA, USA) HemoTypeSC on the first 300 samples at each center for comparison For confirmation, DDE1 PCR and RFLP analysis using Qiagen Flexi Gene DNA kit (Qiagen, Hilden, Germany)
	Prospective study			
	Duration not specified			

HPLC—high-performance liquid chromatography, CDFD—Centre for DNA Fingerprinting and Diagnostics, PCR—polymerase chain reaction, DNA—deoxyribose nucleic acid, RFLP—restriction fragment length polymorphism.

**Table 2:** Estimation of sickle-cell disorder burden in tribal and nontribal populations.

Step	Calculations
Step 1: Calculation of population shares	Tribal population = 8.6% Nontribal = 91.4%
Step 2: Total fertility rate assumptions	Tribal TFR = 2.5 Nontribal TFR = 2.0
Step 3: Calculation of total births in India	Using CBR 16/1000 and population 1.44 billion Total births ≈ 23.04 million
Step 4: Estimating fertility-weighted population share	Tribal = 0.086 × 2.5 = 0.215 Nontribal = 0.914 × 2.0 = 1.828 Total = 0.215 + 1.828 = 2.043
Step 5: Estimating births	Tribal births = (0.215/2.043) × 24.12 ≈ 2.42 million Nontribal births = 24.12 − 2.53 ≈ 20.62 million
Step 6: Extrapolating estimates from this systematic review	Tribal births with sickle cell disorders = 1482 × 24.2 = 35,864 Nontribal births with sickle cell disorders = 5 × 206.2 = 1031 Total births in 2024 with sickle cell disorders = 36,895

## Data Availability

The original contributions presented in this study are included in the Appendix A. Further inquiries can be directed to the corresponding author.

## Abbreviations

The following abbreviations are used in this manuscript.

CBR	crude birth rate
CDFD	Centre for DNA Fingerprinting and Diagnostics
CRHCM	Centre for Research Management and Control of Hemoglobinopathies
DNA	deoxyribose nucleic acid
ED	emergency department
GBD	Global Burden of Disease
HPLC	high-performance liquid chromatography
NAWA	Nilgiris Adivasi Welfare Association
NBS	newborn screening
NFHS	National Family Health Survey
NIRTH	National Institute of Research in Tribal Health
NSCAEM	National Sickle Cell Elimination Program
PCR	polymerase chain reaction
POCT	point-of-care testing
PRISMA	Preferred Reporting Items for Systematic Reviews and Meta-Analyses
RFLP	restriction fragment length polymorphism
SCD	sickle cell disorder
SCT	sickle cell trait
SDG	Sustainable Development Goal
SEWA	Society for Education, Welfare and Action—Rural

## Appendix A

**Table A1:** Search strategy for databases—sickle cell disease in India.

Database	Search Terms
PubMed	(((neonate OR newborn OR infant OR birth) AND screen) OR “early diagnosis”) AND (“sickle cell anemia” OR “sickle cell anaemia” OR “sickle cell disease” OR “sickle trait” OR “sickle cell disorder” OR sickling OR hemoglobinopathy OR “hemoglobin disorder”) AND (India OR Indian)
Embase	(((neonate OR newborn OR infant OR birth) AND screen) OR “early diagnosis”) AND (“sickle cell anemia” OR “sickle cell anaemia” OR “sickle cell disease” OR “sickle trait” OR “sickle cell disorder” OR sickling OR hemoglobinopathy OR “hemoglobin disorder”) AND (India OR Indian)
Scopus	Title-abstract-keyword (((neonate OR newborn OR infant OR birth) AND screen) OR “early diagnosis”) AND (“sickle cell anemia” OR “sickle cell anaemia” OR “sickle cell disease” OR “sickle trait” OR “sickle cell disorder” OR sickling OR hemoglobinopathy OR “hemoglobin disorder”) AND (India OR Indian)
Web of Science	((neonate OR newborn OR infant OR neonatal OR birth OR mass) AND (screen OR screening) OR “early diagnosis”) AND ((“sickle cell” AND (anemia OR anaemia OR disease OR trait OR disorder)) OR sickling OR hemoglobinopath* OR haemoglobinopath* OR “hemoglobin disorder” OR “haemoglobin disorder”) AND (India OR Indian)

**Table A2:** List of excluded studies.

Authors	Title	Journal	Exclusion Reason
Database Search			
Upadhye, 2012	First case of Hb Fontainebleau with sickle haemoglobin and other non-deletional Î± gene variants identified in neonates during newborn screening for sickle cell disorders	Journal of clinical pathology	Case report
Dhingra, 2025	Newborn Screening Implementation: Hurdles, Challenges and Way Forward	Int. J. Neonatal Screen.	Conference abstract, insufficient data
Ambekar, 2001	Pattern of hemoglobinopathies in western Maharashtra	Indian pediatrics	Different population (not newborns)
Andankar, 2025	MDS-952: Mapping the Burden of Sickle Cell Anemia in the Tribal region of Eastern Maharashtra, India: Clinical Profile, Genotypic Patterns, and Spousal Counseling Insights From a 15-Year (2009–2024) Retrospective Study	Clin. Lymphoma Myeloma Leukemia	Different population (not newborns)
Arora, 2001	Prenatal diagnosis of haemoglobinopathies	The National medical journal of India	Different population (not newborns)
Balgir, 2005	Spectrum of hemoglobinopathies in the state of Orissa, India: a ten years cohort study	The Journal of the Association of Physicians of India	Different population (not newborns)
Baruah, 2014	Pattern of hemoglobinopathies and thalassemias in upper Assam region of North Eastern India: high performance liquid chromatography studies in 9000 patients	Indian journal of pathology & microbiology	Different population (not newborns)
Cherian, 2022	Spectrum of Haemoglobinopathies Detected on Antenatal Screening and Diagnostic Work-up in an Urban Healthcare Set-up: A Retrospective Study	Journal of Clinical and Diagnostic Research	Different population (not newborns)
Dash, 2005	Screening for haemoglobinopathies and G6PD deficiency among the Mizos of Mizoram: a preliminary study	Indian journal of pathology & microbiology	Different population (not newborns)
Dhebane, 2023	Hemoglobinopathies in adults and cord blood analysis for neonatal screening with special reference to Alpha Thalassemia—A pilot study from central India	Int. J. Life Sci. Biotechnol. Pharma Res.	Different population (not newborns)
Dipal, 2013	Antenatal Screening for Identification of Couples for Prenatal Diagnosis of Severe Hemoglobinopathies in Surat, South Gujarat	Journal of Obstetrics and Gynecology of India	Different population (not newborns)
Khatua, 2024	Spectrum of Sickle Cell Hemoglobinopathies Using High-Performance Liquid Chromatography as Diagnostic Tool: A Study from Western Odisha, India	SSR Inst. Int. J. Life. Sci.	Different population (not newborns)
Maji, 2020	Implications of Population Screening for Thalassemias and Hemoglobinopathies in Rural Areas of West Bengal, India: Report of a 10-Year Study of 287,258 Cases	Hemoglobin	Different population (not newborns)
Mandot, 2009	Sickle cell anemia in Garasia tribals of Rajasthan	Indian Pediatrics	Different population (not newborns)
Menon, 2011	Community based comprehensive care for sickle cell disease for a remote tribal population in Nilgiri hills, Southern India	Am. J. Hematol.	Different population (not newborns)
Mohanty, 2022	Prevalence of sickle cell anemia, Î^2^-thalassemia and glucose-6-phosphate dehydrogenase deficiency among the tribal population residing in the Aravali hills of Sirohi region of Rajasthan state	Clinical Epidemiology and Global Health	Different population (not newborns)
Nayar, 2017	Spectrum of Haemoglobinopathies: A Hospital Based Study in Uttarakhand	Journal of Clinical and Diagnostic Research	Different population (not newborns)
Raman, 2017	A Study Of Haemoglobinopathies And Haemoglobin Variants Using High Performance Liquid Chromatography (HPLC) In A Teaching Hospital Of Odisha	Journal of Evolution of Medical and Dental Sciences	Different population (not newborns)
Ramasamy, 1994	Prevalence of sickle cells in Irula, Kurumba, Paniya & Mullukurumba tribes of Nilgiris (Tamil Nadu, India)	Indian Journal of Medical Research	Different population (not newborns)
Sachdev, 2010	Detection of Hb variants and hemoglobinopathies in Indian population using HPLC: Report of 2600 cases	Indian Journal of Pathology and Microbiology	Different population (not newborns)
Shrikhande, 2014	Prevalence of the Î^2^(S) gene among scheduled castes, scheduled tribes and other backward class groups in Central India	Hemoglobin	Different population (not newborns)
Singh, 2017	Spectrum of hemoglobinopathies and thalassemias diagnosed on HPLC in a tertiary teaching hospital of Northern India	Indian J. Hematol. Blood Transfus.	Different population (not newborns)
Vachhani, 2022	Spectrum of Î^2^-Thalassemia and Other Hemoglobinopathies in the Saurashtra Region of Gujarat, India: Analysis of a Large Population Screening Program	Hemoglobin	Different population (not newborns)
Yogalakshmi, 2020	Screening for haemoglobinopathies-better prevent than regret-a tip of the ICEBERG experience in a tertiary care hospital in Chennai	Int. J. Res. Pharm. Sci.	Different population (not newborns)
Italia, 2011	Newborn screening for sickle cell disease in a tribal area of south Gujarat, India	Am. J. Hematol.	Duplication of dataset
Jain, 2016	Clinical events in a large prospective cohort of children with sickle cell disease in Nagpur, India: evidence against a milder clinical phenotype in India	Pediatr. Blood Cancer	Duplication of dataset
Patel, 2014	Newborn Screening for Sickle Cell Disease in India: The Need for Defining Optimal Clinical Care	Indian Journal of Pediatrics	Editorial
Jain, 2020	Homozygous sickle cell disease in Central India & Jamaica: A comparison of newborn cohorts	Indian Journal of Medical Research	Only babies born to sickle heterozygous mothers tested
Jain, 2012	Newborn Screening Shows a High Incidence of Sickle Cell Anemia in Central India	Hemoglobin	Only babies born to sickle heterozygous mothers tested
Upadhye, 2019	Red Cell Indices and Hemoglobin Profile of Newborn Babies with Both the Sickle Gene and Alpha Thalassaemia in Central India	Indian Journal of Hematology and Blood Transfusion	Only babies born to sickle heterozygous mothers tested
Upadhye, 2014	Newborn screening for haemoglobinopathies by high performance liquid chromatography (HPLC): diagnostic utility of different approaches in resource-poor settings	Clinical Chemistry and Laboratory Medicine	Only babies born to sickle heterozygous mothers tested
Dave, 2022	Newborn Screening and Clinical Profile of Children With Sickle Cell Disease in a Tribal Area of Gujarat	Indian Pediatrics	Only babies born to sickle heterozygous or homozygous mothers tested
Upadhye, 2016	Neonatal Screening and the Clinical Outcome in Children with Sickle Cell Disease in Central India	Plos One	Only babies born to sickle heterozygous or homozygous mothers tested
Balgir, 2000	The burden of haemoglobinopathies in India and the challenges ahead	Current Science	Review article
Colah, 2015	Sickle cell disease in tribal populations in India	Indian Journal of Medical Research	Review article
Colah, 2018	Newborn screening for sickle cell disease: Indian experience	International Journal of Neonatal Screening	Review article
Colombatti, 2023	Systematic Literature Review Shows Gaps in Data on Global Prevalence and Birth Prevalence of Sickle Cell Disease and Sickle Cell Trait: Call for Action to Scale Up and Harmonize Data Collection	Journal of Clinical Medicine	Review article
Dexter, 2022	Saving lives through early diagnosis: the promise and role of point of care testing for sickle cell disease	British Journal Of Haematology	Review article
Ghosh, 2015	Haemoglobinopathies in tribal populations of India	The Indian Journal Of Medical Research	Review article
Gupta, 2024	Sickle cell disease in India: the journey and hope for the future	Hematology. American Society Of Hematology. Education Program	Review article
Ismail, 2020	Effectiveness of Comprehensive Newborn Screening Program of Sickle Cell Disease on the Childhood Morbidity and Mortality of the Disease: A Systematic Review and Meta-Analysis	Blood	Review article
Mookken, 2020	Universal Implementation of Newborn Screening in India	International Journal of Neonatal Screening	Review article
Mukherjee, 2020	Newborn screening for sickle cell disease in India: Successes and challenges		Review article
Nietert, 2002	Sickle cell anaemia—Epidemiology and cost of illness	Pharmacoeconomics	Review article
Piel, 2013	Global Burden of Sickle Cell Anaemia in Children under Five, 2010–2050: Modelling Based on Demographics, Excess Mortality, and Interventions	Plos Medicine	Review article
Rao, 2024	Prevalence of Sickle cell disease, Sickle cell trait and HBS-beta-thalassemia in India: A systematic review and Meta-analysis	Clinical Epidemiology and Global Health	Review article
Sharma, 2018	Inborn error of metabolism [IEM] screening in neonates	Pravara Medical Review	Review article
Therrell, 2025	Newborn Screening for Hemoglobin Disorders	Clinics in Perinatology	Review article
Verma, 2000	Burden of genetic disorders in India	Indian Journal Of Pediatrics	Review article
Pandey, 2023	NBSP: an online centralized database management system for a newborn sickle cell program in India	Frontiers in Digital Health	Study protocol
Surve, 2025	Protocol for a Multicentric Cohort Study on Neonatal Screening and Early Interventions for Sickle Cell Disease Among High-Prevalence States of India	Diagnostics	Study protocol
Rajamani, 2025	Barriers, facilitators and recommendations for the implementation of newborn sickle cell screening program in tribal communities: findings from a qualitative multicentric study in India	Lancet Regional Health—Southeast Asia	Outside the outcome of this study
Raveendran, 2022	Need and Viability of Newborn Screening Programme in India: Report from a Pilot Study	International Journal Of Neonatal Screening	Outside the outcome of this study
Sharma, 2024	Sickle cell disease in Indian tribal population: Findings of a multi-centre Indian SCD registry	Blood Cells, Molecules & Diseases	Outside the outcome of this study
Surve, 2023	Challenges in screening for sickle cell disease among newborns from the tribal region of Palghar, Maharashtra during the COVID-19 pandemic	Indian Journal of Medical Research	Outside the outcome of this study
**Other Sources**			
Abhilash Gajula, 2015	The Relevance of Newborn Screening to India: A Systematic Review Newborn Screening in the Indian Setting: Recommendations and Challenges	Masters’ dissertation	Review
Rakesh Jha, 2023	The Neonatal Screening for Sickle Cell Disease, Thalassemia, and G6PD Deficiency in Central India	Conference Abstract	Conference abstract
Aishwarya Arjunan, 2013	A Systematic Evaluation of a Newborn Screening Program for Sickle Cell Disease In Southern Gujarat, India	Masters’ dissertation	Outside the outcome of this study
Pradeep, 2011	Screening for the sickle cell gene in Chhattisgarh state, India: an approach to a major public health problem	J Community Genet	Different population (not newborns)
Patra, 2015	The Chhattisgarh state screening programme for the sickle cell gene: a cost-effective approach to a public health problem	J Community Genet	Different population (not newborns)
Yogita Sharma, 2024	Sickle cell disease in Indian tribal population: Findings of a multi-centre Indian SCD registry	Blood Cells, Molecules And Diseases	Different population (not newborns)
